# 

**Published:** 1857-03

**Authors:** 


VOL. VI. NEW SERIES. No. 3.

### THE NORTH-WESTERN MEDICAL AND SURGICAL JOURNAL.

PUBLISHED MONTHLY.

AT TWO DOLLARS PER ANNUM, IN ADVANCE.

EDITED BY

N. S. DAVIS, M.D.,

PROFESSOR OF PRINCIPLES AND PRACTICE OF MEDICINE AND CLINICAL MEDICINE IN RUSH MEDICAL COLLEGE; ONE OF THE PHYSICIANS TO THE MERCY HOSPITAL; FELLOW OF THE COLLEGE OF PHYSICIANS AND SURGEONS, NEW YORK; MEMBER OF THE MEDICAL SOCIETY OF THE STATE OF NEW YORK, AND OF THE STATE OF ILLINOIS; MEMBER OF THE AMERICAN MEDICAL ASSOCIATION, &C. &C.

MARCH, 1857.

CHICAGO:
ROBERT FERGUS, PRINTER,
189 LAKE STREET.

VOL. XIV. WHOLE SERIES. No. 3.

